# Efficacy evaluation of pneumatic solid set canopy delivery system to control powdery mildew (Erysiphe necator) in Washington vineyards

**DOI:** 10.1002/ps.70596

**Published:** 2026-02-03

**Authors:** Dattatray G Bhalekar, Eric Mozzanini, Ramesh K Sahni, Michelle M Moyer, Lav R Khot

**Affiliations:** ^1^ Center for Precision and Automated Agricultural Systems, Department of Biological Systems Engineering Washington State University Prosser WA USA; ^2^ Department of Agricultural, Forest and Food Sciences (DiSAFA) University of Turin (UNITO) Turin Italy; ^3^ Agricultural Mechanization Division, ICAR‐Central Institute of Agricultural Engineering Bhopal MP India; ^4^ Department of Viticulture and Enology Washington State University Prosser WA USA

**Keywords:** biological efficacy, crop protection, fixed spray delivery system, powdery mildew, vineyards

## Abstract

**BACKGROUND:**

In this study, a previously optimized pneumatic spray delivery (PSD)‐based solid set canopy delivery system (SSCDS) was compared with an airblast sprayer (grower control [GC]) for the delivery of fungicides in the management of powdery mildew (*Erysiphe necator*) in vineyards. For 2023 and 2024 growing seasons, spray coverage was quantified for each application date and treatment. Visual disease severity on clusters and leaves was assessed five times per season. The accumulated area under the disease progression curve (AUDPC) was developed from these ratings.

**RESULTS:**

Over two seasons, spray coverage for PSD‐SSCDS treatment ranged from 16.7% to 32.7%, whereas GC achieved coverage between 39.9% and 62.9%. The average difference in maximum cluster and foliar disease severity between GC and PSD‐SSCDS was 5.5% and 14.2%, respectively.

**CONCLUSIONS:**

Despite lower spray coverage in PSD‐SSCDS, the accumulated AUDPC for cluster disease severity was similar to GC treatments in both growing seasons. However, foliar disease severity differed significantly, with GC showing less disease than PSD‐SSCDS. These study findings indicate that optimal emitter selection is crucial for achieving enhanced spray performance and effective disease control using the PSD‐SSCDS technology in vineyards. Both fungicide spray treatments effectively protected clusters from powdery mildew, indicating PSD‐SSCDS as an emergent alternative spray technology. © 2026 The Author(s). *Pest Management Science* published by John Wiley & Sons Ltd on behalf of Society of Chemical Industry.

## Graphical abstract

The findings of this study indicate that powdery mildew control achieved with the pneumatic solid set canopy delivery system (PSD‐SSCDS) was comparable with that of a conventional airblast sprayer. These results highlight the PSD‐SSCDS as a promising alternative spray technology.

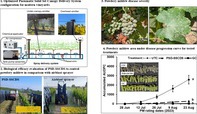

## 1 INTRODUCTION

Washington State is the second‐largest producer of grapes in the USA, with a total grape production of 302 550 tons in 2024.^1^ Disease management is critical in vineyard cultivation, accounting for up to 20% of the production cost.^2^ Powdery mildew, caused by *Erysiphe necator*, is one of the major diseases impacting grape production globally.^3^ Almost all *Vitis vinifera* cultivars, the primary cultivars used for wine production, are susceptible to *E. necator*, which infects all tissues, including leaves, shoots, tendrils, flowers, and berries. Severe foliar infection could reduce photosynthetic capacity significantly and may lead to premature defoliation.^4^ Severe fruit infections can include berry shrivel or cracking, which can lead to secondary infections by fruit‐rotting fungi. Overall, infection by *E. necator* can reduce vine yield and degrade fruit quality, which may result in off‐flavors in the wine.^5^ To control *E. necator*, grape growers implement cultural practices along with a preventative fungicide program.^6^ To reduce humidity in the canopy, a common environmental factor that favors pathogen reproduction, grape growers often implement cultural methods that include removing diseased leaves and shoots, thinning the vine canopy, to ensure sufficient ventilation and sunlight penetration.^7^ Fungicide applications, the primary method for disease control, are beginning to lose their efficacy because of the increased emergence and incidence of fungicide resistance in *E. necator*.^8^

Axial and multi‐axial fan airblast sprayers are commonly used application technologies for crop protection products in Washington vineyards.^9^ However, excess air can result in up to 50% off‐target chemical drift during vineyard applications.^10^ Viret *et al*.^10^ implemented a season‐long chemical spray program to control *E. necator* using a standard axial fan airblast sprayer, tunnel sprayer, high‐volume airblast sprayer, over‐row sprayer, motorized knapsack mist blower, and helicopter sprayer. The study reported improved and comparable disease control using all the tested spray technologies, except for the helicopter and high‐volume airblast sprayers. Those two sprayers also had the highest ground and aerial drift. Chemical drift from agricultural sprayers can result in environmental contamination and increase chemical exposure hazards for humans and animals.

Technological advancements are being made toward developing and evaluating precision chemical application solutions.^11^, ^12^, ^13^ One such tool is the solid set canopy delivery system (SSCDS), a variant of the fixed spray system (FSS). This tool could be a viable crop protection technology for specialty crops in the USA^14^, ^15^ and European countries.^16^, ^17^ Sinha *et al*.^18^ reported that SSCDS can significantly reduce off‐target drift compared to conventional airblast sprayers in Washington vineyards, demonstrating its potential as a chemical‐saving spray technology. Innerebner *et al*.^16^ evaluated the biological efficacy of fungicide treatments in vineyards to control downy and powdery mildew applied using the FSS prototype and compared that with an airblast sprayer and saw no differences in fruit disease control. However, reduced foliar disease control in FSS compared with an airblast sprayer was observed during weather conditions favorable for downy mildew (*Plasmopara viticola*) infections. Imperatore *et al*.^19^ conducted a season‐long biological efficacy evaluation of crop protection products applied via an FSS, airblast sprayer, as well as spray gun, and found no differences between application approaches for the management of grapevine powdery mildew, downy mildew, and other pests. Owen‐Smith *et al*.^20^ evaluated SSCDS and a conventional airblast sprayer to control oblique banded leafroller (OBLR) in apples and reported 95% and 98% larval mortality compared with the untreated control (UTC), respectively. Sahni *et al*.^21^ conducted a bioassay‐based evaluation of a pneumatic spray delivery‐based SSCDS (PSD‐SSCDS) to control OBLR and codling moth (CM) in modern apple orchards. The study reported 91% and 98% larval mortality of OBLR for pneumatic SSCDS and airblast sprayer treatments, respectively. Larval CM mortality was 100% in both treatments compared with the UTC.

Based on the mode of propeller, either water or compressed air, SSCDS has been categorized as either hydraulic spray delivery based (HSD)^17^ or PSD^21^ based. Bhalekar *et al*.^22^ optimized the emitter configuration in a PSD‐SSDCS for vertical shoot positioning (VSP) vineyards and saw improved spray deposition (by 78%) and coverage (by 40%), compared with previously optimized HSD‐SSCDS.^23^ Moreover, the overall PSD‐SSDCS cost was reduced by 54% compared with HSD‐SSCDS; this was done by reducing emitter cost (by 66%) and emitter density per hectare (by 37%), However, cost and spray deposition alone do not drive large‐scale adoption of such a system; fundamentally, the spray system will need to provide equivalent or better control of critical pests and diseases in the target crop. The objective of the study presented here was to evaluate whether a season‐long fungicide program applied using a PSD‐SSCDS provided comparable control of *E. necator* relative to a conventional airblast sprayer in a *V. vinifera* vineyard.

## 2 MATERIALS AND METHODS

### 2.1 Experimental site

Trials for PSD‐SSCDS based control of *E. necator* were performed in an experimental *V. vinifera* vineyard (cv. Chardonnay) at the Washington State University‐Irrigated Agriculture Research and Extension Center, Prosser, WA, USA (46° 15′ 8.39″ N, 119° 44′ 24.47″ W) during the two growing seasons (2023 and 2024). The vineyard block was planted in 2009 and is not grafted onto a rootstock. The vines were spaced 1.8 m apart, with 3.0‐m row spacing. The trellis system was a modified vertical shoot position (mVSP) system, with only three support wires and no movable catch wires. The vines were trained to a dual‐trunk, bilateral cordon, and spur‐pruned to two‐bud spurs in the late winter preceding each growing season. The vineyard was drip‐irrigated and featured natural vegetation between the rows and under the vines. Routine irrigation, fertilization, mowing, and canopy and trunk management of shoots were performed during both study seasons.

### 2.2 Experimental design

**Figure 1:**
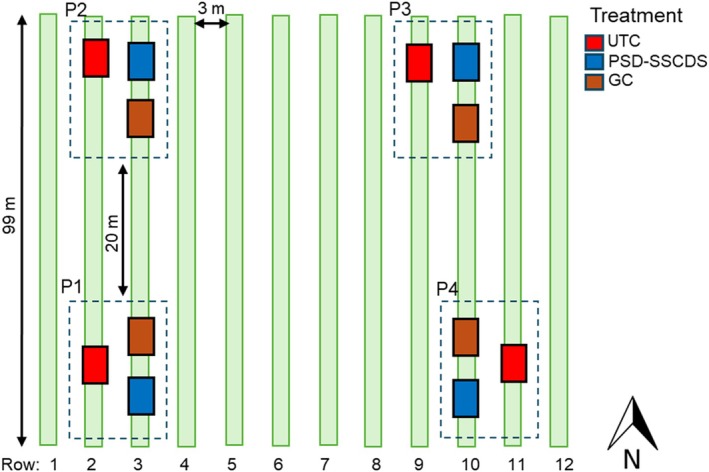
Field layout of treatments: untreated control (UTC), pneumatic solid set canopy delivery system (PSD‐SSCDS), and grower control (GC) in the experimental vineyard (schematic not drawn to scale). The same layout was used each year of the study.

The efficacy of PSD‐SSCDS to control *E. necator* was compared with a conventional airblast sprayer [hereafter referred to as grower control (GC]) and unsprayed control (hereafter referred to as UTC) treatment. Each treatment was replicated four times using a randomized block design in an experimental vineyard (Fig. 1). Five vine rows were randomly selected and divided into four plots [P1–P4, 0.3 ha (99 m × 30 m]) with a minimum buffer of 18 m among the plots. Within each plot, the three treatments (PSD‐SSCDS, GC, UTC) were randomly assigned, as illustrated in Fig. 1. Each treatment was replicated four times within the block, with six vines per replicate.

### 2.3 Spray system

**Figure 2:**
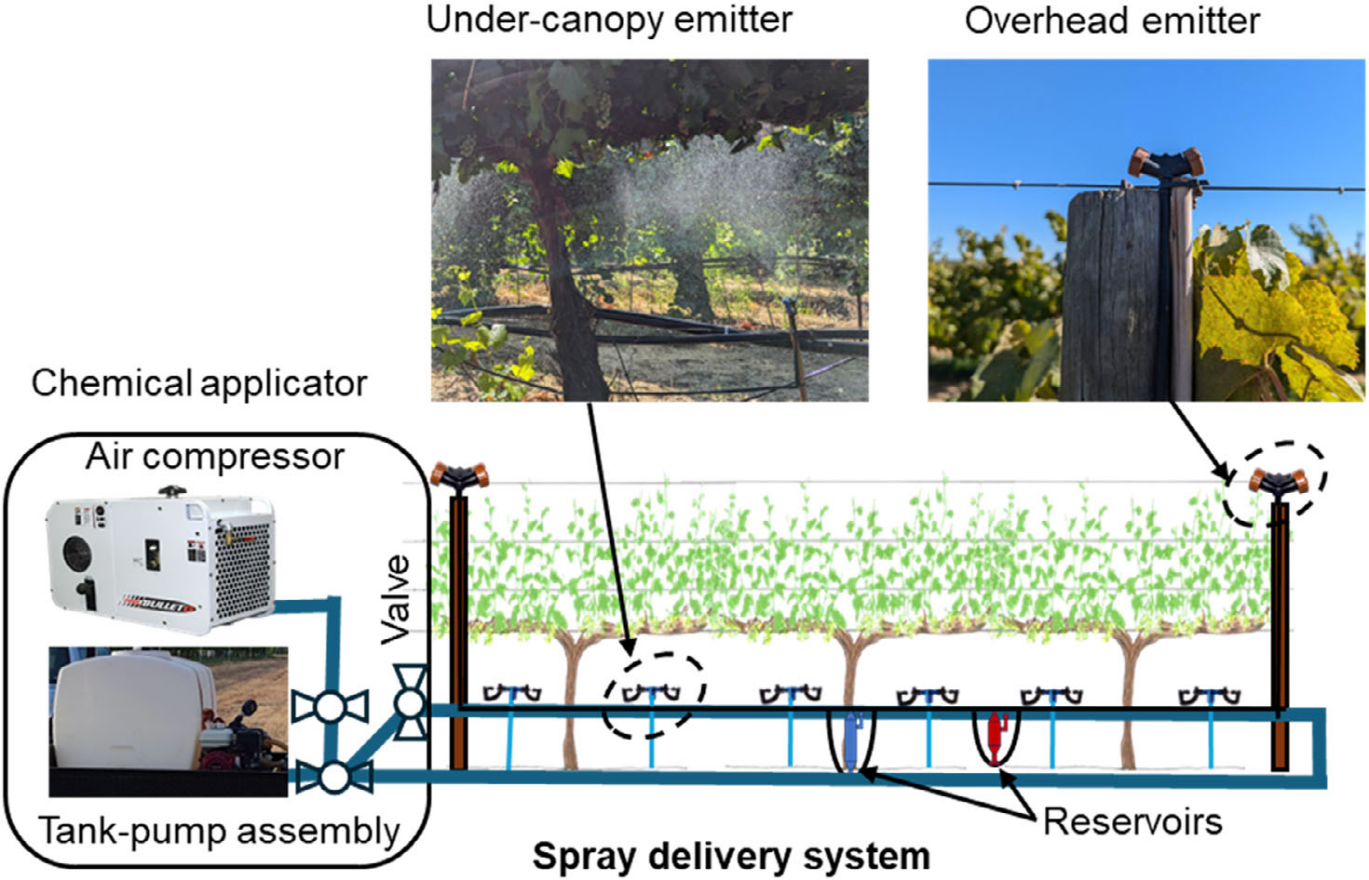
Schematic representation of optimized pneumatic solid set canopy delivery system for vineyards (schematic not drawn to scale).

**Figure 3:**
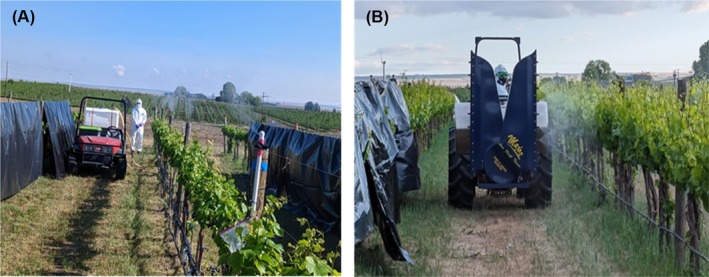
Field images of pneumatic solid set canopy delivery system (A) and grower control treatment (B) using an airblast sprayer during fungicide sprays.

The PSD‐SSCDS configuration previously optimized by Bhalekar *et al*.^22^ (Fig. 2) was installed in this trial. Each PSD‐SSCDS replicate consisted of chemical reservoirs designed to spray 234 L ha^−1^ per spray cycle through the emitters installed in overhead (Modified StripNet STR31, Netafim Ltd, Israel) and under‐canopy (modified 90° modular Flat Fan, Jain by Rivulis Inc., Fresno, CA, USA), source line, and return line covering nine vines (16.2 m) (Fig. 3(A)). Chemical reservoirs R1 (350 mL) and R2 (235 mL) were installed at a 5.5‐m row length spacing to evenly spray the chemical volume in the top and bottom canopy zones, respectively. The source lines were installed on existing trellis wire for drip irrigation lines (0.6 m above ground level). Typically, PSD‐SSCDS is installed in a loop with a return line passing through adjacent rows; however, in this study, the return lines were laid on the ground, because installation was limited to a single row.

A mobile chemical applicator unit was used to perform spray applications, which consisted of an air compressor (Bullet G 70, Boss Industries, LLC, LaPorte, IN, USA), a tank (capacity 378.5 L), and a centrifugal pump (model 1538, Hypro, New Brighton, MN, USA). Typical to existing PSD‐SSCDS, each spray cycle consisted of: (i) the chemical mixture filling (hydraulic pressure: 69 kPa) and recovery (compressed air: 69 kPa); (ii) followed by spraying and cleaning using compressed air at 310 kPa.

For the GC treatment, a conventional tractor (model T4.75, New Holland Agriculture Ltd, New Holland, PA, USA) operated axial fan tower‐shaped airblast sprayer (model S24P200, low‐drift tower, TurboMist by Slimline Mfg., Penticton, BC, Canada). The sprayer was equipped with TeeJet VisiFlo TX‐VK12 nozzles (TeeJet Technologies, Glendale Heights, IL, USA) (Fig. 3(B)).

### 2.4 Fungicide applications

Fungicide treatments, in both years, began at vine development stage of BBCH 16 (six leaves unfolded)^24^ and continued at either weekly or bi‐weekly intervals. The late‐season spray schedule was adjusted by up to 2 days because of weather conditions. The standard fungicide program, designed using sulfur‐based and synthetic chemicals for *E. necator* control specific to Washington vineyards,^25^, ^26^ was followed in this study (Table 1). Owing to low canopy volume, the application rates of 234 L ha^−1^ and 341 L ha^−1^ were sprayed in PSD‐SSCDS and GC (four nozzles per side) treatments on 15 May 2024, respectively. However, all the remaining sprays for both treatments were applied at a rate of 468 L ha^−1^ (five nozzles per side in GC). Polyethylene tarps were used to cover individual treatment replicates during each spray to reduce potential drift between treatment plots.

**Table 1:** Fungicide spray regime followed in this study to control powdery mildew in 2023 and 2024 seasons using both pneumatic solid set canopy delivery system and grower control treatments

Spray event	Spray dates (BBCH^†^)		Product trade names used^‡^ (ha^−1^)	
	2023	2024	2023	2024
1	17 May (16)	15 May (16)	Microthiol Disperss (4.48 kg), Nufilm P (0.29 L)	Microthiol Disperss (3.36 kg), Nufilm P (0.29 L)
2	24 May (57)	22 May (57)	Microthiol Disperss (3.36 kg), Quintec (0.48 L)	Microthiol Disperss (3.36 kg), Nufilm P (0.29 L)
3	6 June (65)	29 May (61)	Vivando (1.12 L), Nufilm P (0.29 L)	Microthiol Disperss (3.36 kg), Quintec (0.48 L)
4	21 June (75)	12 June (65)	Torino (0.25 L), Nufilm P (0.29 L)	Torino (0.25 L), Nufilm P (0.29 L)
5	7 July (77)	26 June (71)	Aprovia (0.77 L), Microthiol Disperss (2.25 kg), Nufilm P (0.29 L)	Vivando (1.12 L), Nufilm P (0.29 L)
6	18 July (79)	12 July (75)	Microthiol Disperss (4.48 kg), Nufilm (0.29 L)	Aprovia (0.77 L), Microthiol Disperss (3.36 kg), Nufilm P (0.29 L)
7	—	25 July (79)	—	Microthiol Disperss (4.48 kg), Nufilm P (0.29 L)

^†^ BBCH is a coding system that standardizes the stages of grapevine development.^24^

^‡^ All chemicals were registered for use as fungicides in Washington vineyards.

### 2.5 Spray coverage quantification

**Figure 4:**
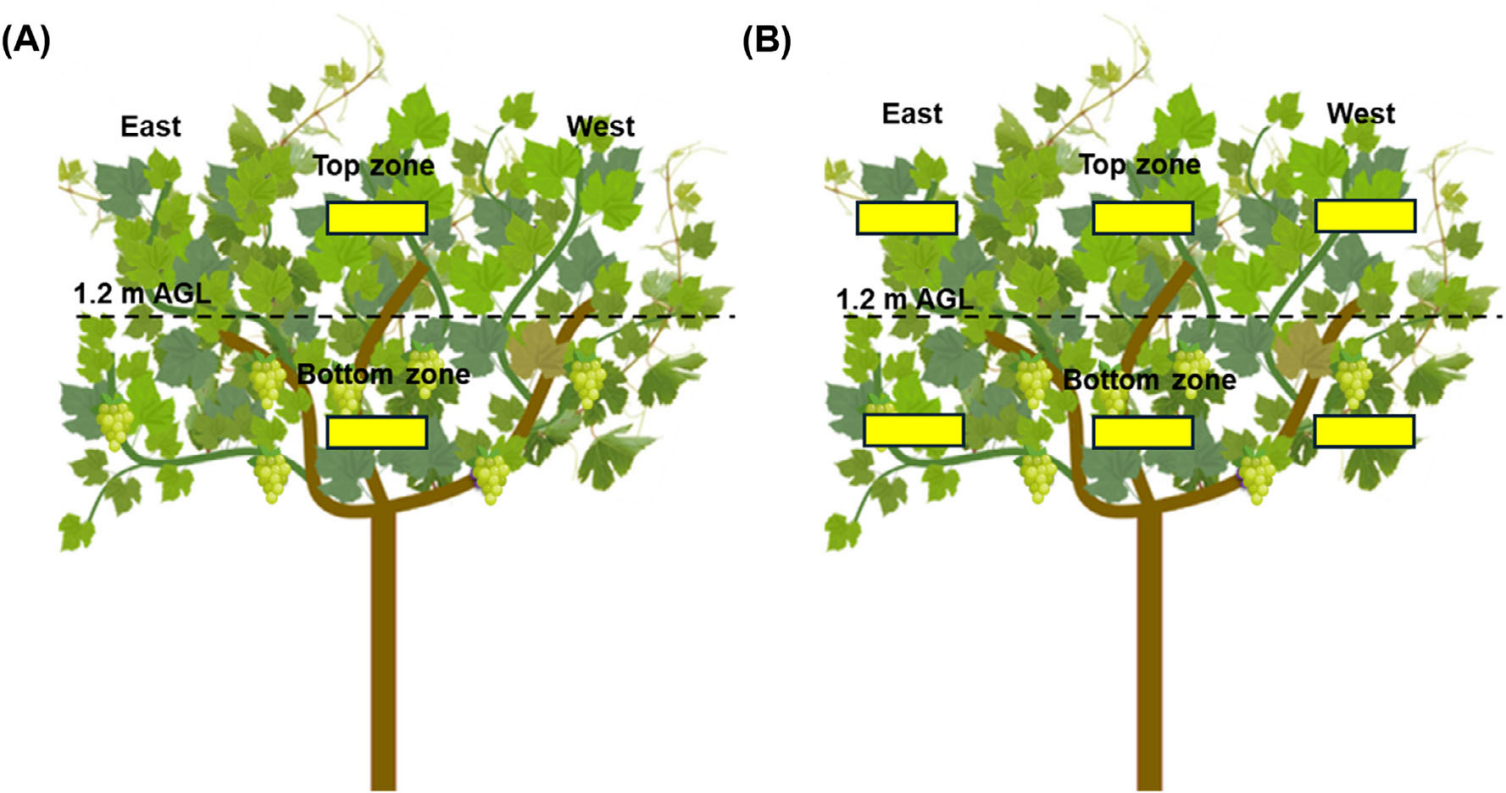
Schematic representation of water‐sensitive paper samplers placement in canopy for mid (A) and late (B) growth stages in the 2023 season, and late growth stage in the 2024 season (B).

Per each PSD‐SSCDS and GC treatment replicate, three vines were randomly selected for spray coverage quantification. Each selected vine canopy was divided into the bottom (0.91–1.47 m) and top canopy zone (1.47–2.02 m). To quantify the spray coverage across treatments, water‐sensitive paper (WSP, 2.54 cm × 10.16 cm; Syngenta Crop Protection Inc., Greensboro, NC, USA) was placed within the vine canopy on both the adaxial and abaxial leaf surfaces (Fig. 4). The placement of WSP in the canopy differed as the season progressed and was further altered for the late season in 2024 (Table 2). Following each spray, the WSP was allowed to dry for 15 min in the field, then collected and stored in a dry location until image analysis.

**Table 2:** Summary of water‐sensitive papers (WSP) based spray coverage assessment during trials in two seasons

Year	Spray event (season)	No. of WSP per spray event	Set of WSP used within the canopy
2023	1–2 (early)	48	2 (Treatments) × 4 (replicates/treatment) × 3 (vines/ replicate) × 2 (leaf surfaces/vine)
	3–6 (mid‐late)	96	2 (Treatments) × 4 (replicates/treatment) × 3 (vines/ replicates) × 2 (zones/vine) × 2 (leaf surfaces/zone)
2024	1–2 (early)	48	2 (Treatments) × 4 (replicates/treatment) × 3 (vines/ replicate) × 2 (leaf surfaces/vine)
	3–4 (mid)	96	2 (Treatments) × 4 (replicates/treatment) × 3 (vines/ replicate) × 2 (zones/vine) × 2 (leaf surfaces/zone)
	5–7 (late)	288	2 (Treatments) × 4 (replicates/treatments) × 3 (vines/ replicates) × 3 (sides/vine) × 2 (zones/side) × 2 (leaf surfaces/zone)

### 2.6 Canopy characterization

Vine canopy growth in each spray treatment replicate was characterized for each spray event by measuring canopy height and width. Tree row volume (TRV, m^3^ ha^−1^) was calculated by multiplying canopy height by the width of randomly selected vines within each treatment replicate in 2023 and 2024, respectively. For sprays during the mid‐growth stage in 2023 season (6 June), excessive canopy growth resulted in unsprayed areas in PSD‐SSCDS treatment. Thus, shoots in pertinent replicate plots were tucked within trellis wires before 21 June application to overcome this issue. Based on this experience, and to improve spray penetration, light canopy management was performed in all the treatments during the 2024 season.

### 2.7 Weather data

An all‐in‐one weather station (ATMOS 41, Meter Group, Pullman, WA, USA) was installed at 2.83 m AGL (1 m above the canopy) to monitor weather parameters during spray trials. The station was connected to a data logger (CR1000X, Campbell Scientific Inc., Logan, UT, USA) to record air temperature (°C), wind speed (m s^−1^), relative humidity (RH; %), and wind direction (°) at every 15 s. All spray applications were performed in the morning hours between 05:00 and 09:00 h [Pacific Daylight Time (PDT]) to avoid negative impact of unsuitable weather conditions on fungicide sprays. The weather conditions monitored at the time of spraying are presented in Table 3.

**Table 3:** Weather data (mean ± SD) monitored during spray applications using a field deployed weather station

Year	Spray dates	Time (PDT*)	Air temperature (°C)	Relative humidity (%)	Wind speed (m s^−1^)	Wind direction (°)
2023	17 May	06:00 to 08:57	15.38 ± 1.66	99 ± 2	1.26 ± 0.57	231.90
	24 May	06:00 to 08:32	11.56 ± 1.49	70 ± 6	1.05 ± 0.49	188.75
	6 June	06:00 to 07:39	17.19 ± 2.88	49 ± 6	2.29 ± 0.71	228.79
	21 June	06:00 to 07:42	11.34 ± 1.79	77 ± 6	1.14 ± 0.68	201.49
	7 July	05:30 to 07:31	17.54 ± 1.83	59 ± 4	1.64 ± 0.35	304.72
	18 July	05:30 to 07:59	13.93 ± 2.80	70 ± 10	1.70 ± 0.5	315.85
2024	15 May	06:10 to 08:15	13.45 ± 2.91	60 ± 10	1.14 ± 0.5	63.47
	22 May	06:00 to 08:45	9.86 ± 0.82	60 ± 10	0.93 ± 0.5	244.54
	29 May	06:00 to 08:30	10.55 ± 2.42	70 ± 10	1.63 ± 1.1	297.77
	12 June	06:00 to 08:30	11.79 ± 2.22	60 ± 10	1.96 ± 0.7	289.68
	26 June	06:00 to 08:25	19.28 ± 1.54	60 ± 0	1.14 ± 0.5	301.37
	12 July	05:00 to 07:52	17.18 ± 3.29	60 ± 10	1.44 ± 0.8	33.61
	25 July	06:30 to 08:33	14.11 ± 1.93	70 ± 10	1.45 ± 0.9	276.96

* PDT, Pacific Daylight Time.

**Figure 5:**
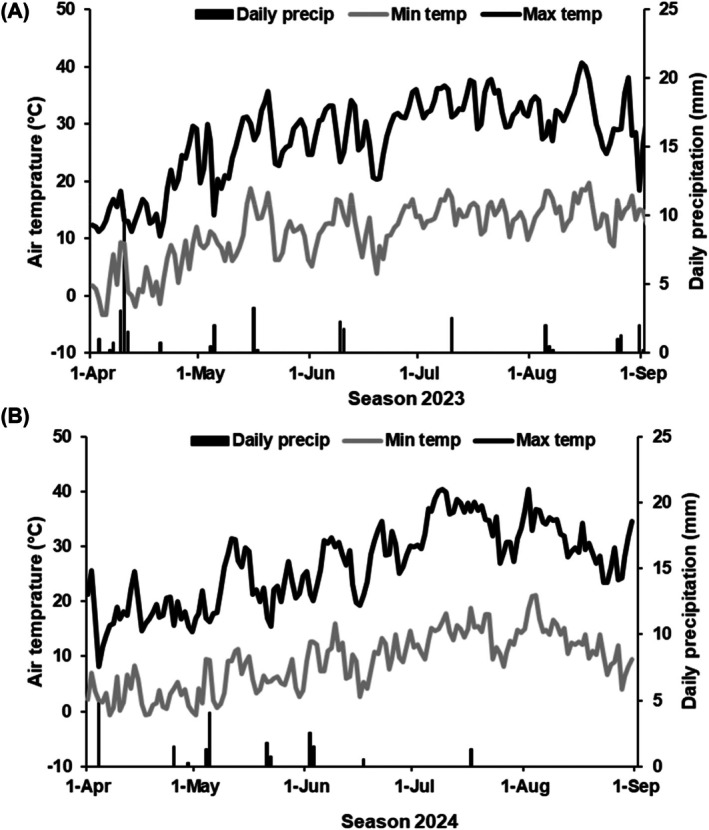
Weather variations during seasons 2023 (A) and 2024 (B). Source: Washington State University's AgWeatherNet ‘Prosser NE’ weather station.

In addition, data from a local weather station (‘Prosser NE’, WSU AgWeatherNet, Prosser WA, USA; https://weather.wsu.edu) located 1.6 km from the experimental vineyard were used to understand seasonal trends. These data include daily precipitation and maximum and minimum air temperature (Fig. 5(A), (B)).

Unfavorable weather patterns, which could hinder *E. necator* development, were observed at the study site during both seasons, characterized by slightly above‐average air temperatures (>35 °C) and below‐average precipitation (rainfall <164 mm). The maximum daily air temperature exceeded 35 °C for 21 and 22 days, from bloom to harvest, in the 2023 and 2024 seasons, respectively. These weather conditions are common in eastern Washington.

### 2.8 Powdery mildew disease incidence and severity assessment

**Figure 6:**
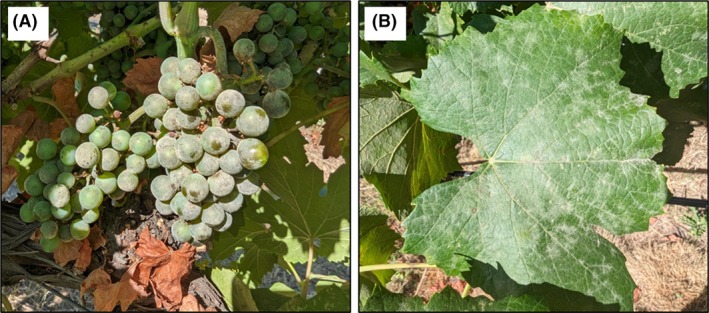
Visual assessment of powdery mildew (*Erysiphe necator*) severity on clusters (A) and leaves (B).

Powdery mildew disease severity on clusters (Fig. 6(A)) and leaves (Fig. 6(B)) were visually assessed as the percentage of leaf surface infected by *E. necator*. For each treatment replicate, 40 clusters or leaves were rated. Assessments were made at 14‐day intervals during growth stages BBCH 75 to 81, for a total of five assessment points per season. The same two trained raters were used for all assessments throughout the season for consistency in disease ratings.

At the end of the season, 30 clusters per treatment replicate were harvested. The clusters were collectively weighed to determine the average cluster weight (g). From each set of 30 clusters, 100 berries were removed and collectively weighed to determine berry weight (g).

### 2.9 Data analysis

To analyze spray coverage, WSP were scanned using a document scanner (Epson Perfection V37, Seiko Epson Corporation, Suwa, Japan; 600 dpi resolution). The digitized documents were exported in image (jpg) format and processed using ImageJ (v.1.52n, Wayne Rasband, NIH, Bethesda, MD, USA) to quantify the spray coverage (%). The raw coverage data were strategically organized and normalized using a cube‐root transformation. A two‐sample *t*‐test was performed on the overall spray coverage and TRV data set to identify significant treatment differences. To better understand the spray coverage distribution trends within the canopy zones and sides, analysis of variance (ANOVA) was performed for mid‐ and late‐season spray events only. Statistical analysis was conducted on the coverage and TRV data set for each spray event separately, and statistical significance was assessed using Tukey's honest significant difference test.

The area under the disease progress curve (AUDPC) values were calculated using Eqn (1) as described by Madden *et al*.,^27^

(1) $$\text{AUDPC}=\sum_{i=1}^{n-1}\left[\left(y_{i}+y_{i+1}\right)/2\right]\times \left(t_{i+1}-t_{i}\right)$$

Where, *y*_*i*_ is the averaged disease severity ratings (%) at *i*th rating, *t*_*i*_ is the rating interval, and *n* is the total number of ratings. The disease progression throughout the growing season was visualized using accumulated AUDPC at each rating event. The linear mixed model was fitted using the restricted maximum likelihood (REML) method to analyze accumulated AUDPC, cluster and berry weight. The model considered treatments as fixed and replicates as random effects. Post‐hoc Tukey's honest significant difference (HSD) test was performed on the REML model, and statistical significance was interpreted at a 5% significance level. All the statistical analysis was performed in RStudio software (v.3.6.0, RStudio, R Team, Vienna, Austria).

## 3 RESULTS

### 3.1 Overall spray coverage

**Figure 7:**
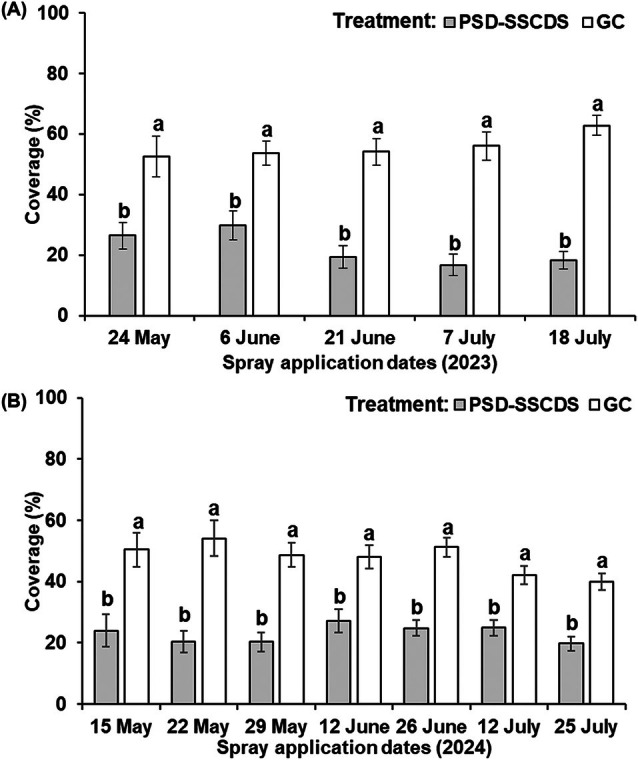
Overall spray coverage quantified for season‐long fungicide spray applications using pneumatic solid set canopy delivery system (PSD‐SSCDS), and grower control (GC) treatments in the 2023 (A) and 2024 (B) seasons. Distinct letters indicate statistically significant differences between treatment means on a given date at *α* = 0.05, as represented by Tukey's honest significant difference test.

Spray coverage between PSD‐SSCDS and GC was statistically different for all the spray events in both seasons (Table 4). During the 2023 season, the spray coverage (mean ± SE) in PSD‐SSCDS was consistently lower than in the GC treatment for all spray dates (Fig. 7(A)). Coverage data for the first spray (17 May 2023) were discarded because of excessive humidity during the spray (Table 3). For early‐season sprays, on 24 May, the spray coverage for PSD‐SSCDS was significantly less than for GC (*P* < 0.0001). Similar trends were observed for mid‐season sprays (6 and 21 June), with the canopy in the PSD‐SSCDS treatment receiving lower coverage in GC (*P* < 0.0001). The coverage differences between the two treatments increased as the season progressed. The lowest spray coverage of the season was observed in PSD‐SSCDS on 7 July (16.70%), followed by 18 July (18.24%). By contrast, GC provided significantly higher spray coverage (*P* < 0.0001) of 56.10% and 62.86% during the respective spray events.

**Table 4:** Comparison of effects on spray coverage

Year	Date	Treatment	Zone	Side†	Treatment: Zone	Treatment: Side	Zone: Side	Treatment: Zone: Side
2023	6 June	< 0.0001	0.195		0.249			
	21 June	< 0.0001	0.031		0.668			
	7 July	< 0.0001	0.021		0.920			
	18 July	< 0.0001	0.560		0.155			
2024	29 May	< 0.0001	0.523		0.107			
	12 June	< 0.0001	0.159		0.923			
	26 June	< 0.0001	0.350	0.656	0.169	0.538	0.846	0.093
	12 July	< 0.0001	0.218	0.416	0.655	0.205	0.322	0.225
	25 July	< 0.0001	0.542	0.186	0.446	0.116	0.883	0.580

^†^ Coverage across canopy sides was quantified only for the last three sprays in season 2024.

Analysis of variance (*P*‐values) conducted on cube‐root transformed spray coverage data set for treatment, canopy zone, and side as main and interaction effects.

A similar trend was observed in the 2024 season, during which GC consistently had higher spray coverage than PSD‐SSCDS across early‐ (*P* = 0.0005), mid‐ (*P* < 0.0001), and late‐season (*P* < 0.0001) spray events (Fig. 7(B)). The highest overall spray coverage was obtained in GC on 22 May (54.06%), followed by June 26 (51.26%); the lowest overall coverage was quantified in PSD‐SSCDS on 18 July (19.78%).

### 3.2 Spray coverage distribution within the canopy

**Figure 8:**
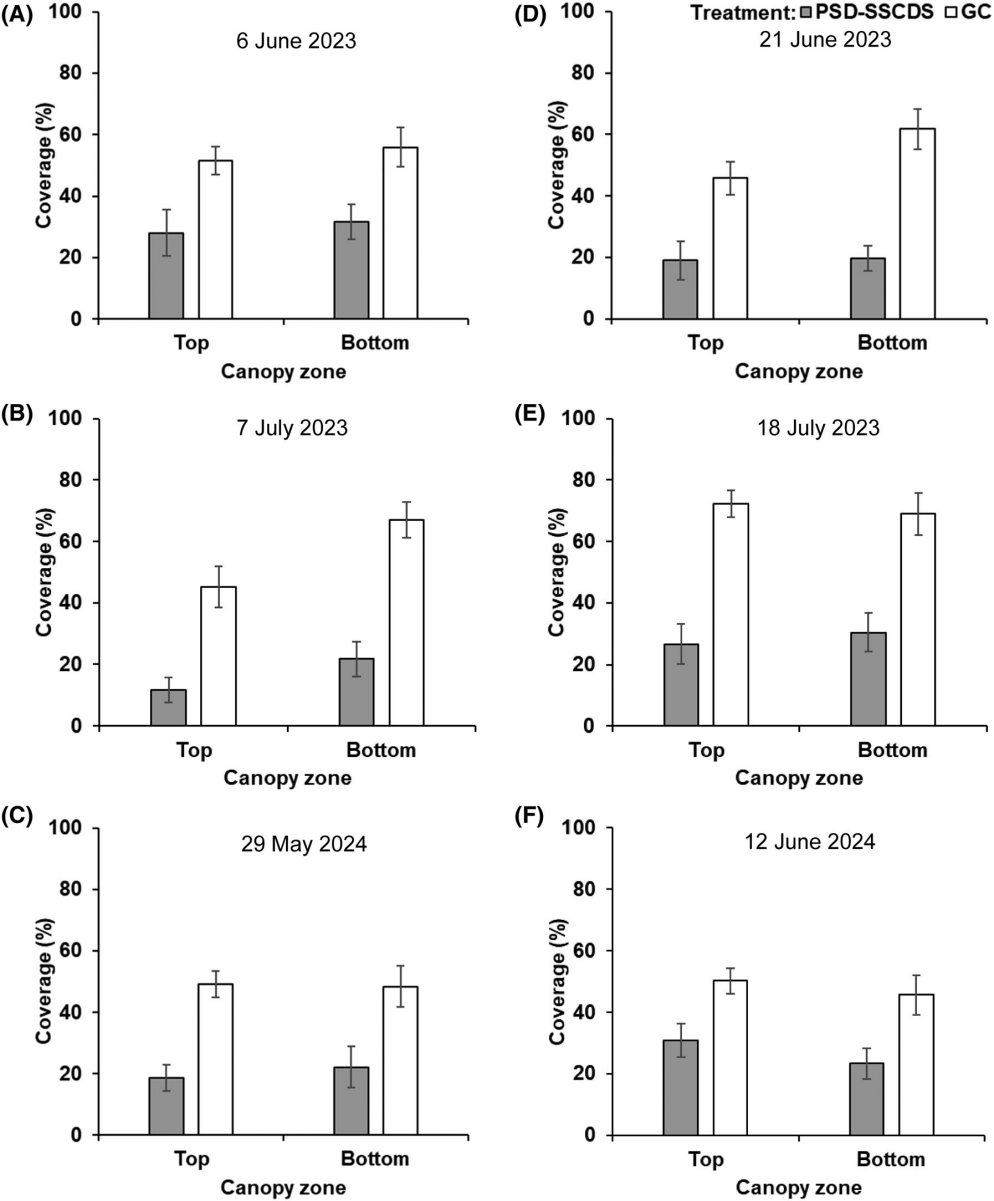
Spray coverage variations in tested spray treatments for spray events conducted on 6 June (A), 21 June (D), 6 July (B), and 18 July (E), in 2023 season, as well as on 29 May (C) and 12 June (F) in 2024 season for pneumatic solid set canopy delivery system (PSD‐SSCDS) and grower control (GC) treatment. Mean coverage and standard errors are represented by vertical bar plots and error bars, respectively.

Interestingly, the canopy zone factor did not influence spray coverage, except on 21 June (*P* = 0.031) and 7 July (*P* = 0.021) in 2023. Significantly lower spray coverage was quantified in both top (18.95%) and bottom (19.63%) canopy zones in PSD‐SSCDS compared with the respective zones in GC (45.72% and 61.75%) on 21 June (Fig. 8). A similar zonal coverage trend was observed on 7 July, with the least quantified coverage in the top zone (11.67%) of the PSD‐SSCDS treatment. There were no interactive effects between treatment and zones across all mid‐ and late‐season sprays (Table 4).

**Figure 9:**
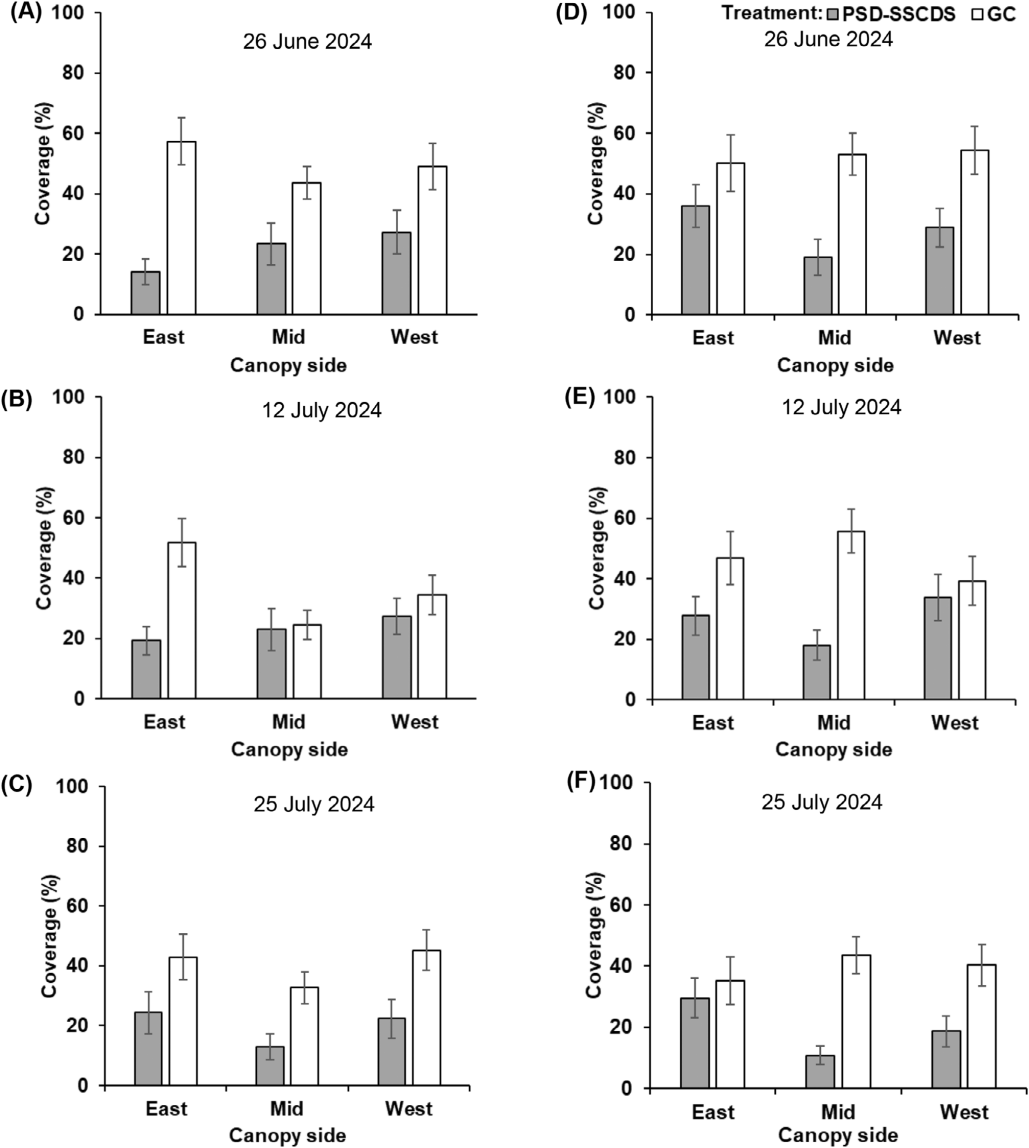
Spray coverage variations in top zone (A, B, C), and bottom (D, E, F) zones for spray events conducted on 26 June (A, D), 12 July (B, E), and 25 July (C, F) in 2024 season for pneumatic solid set canopy delivery system (PSD‐SSCDS) and grower control (GC) treatment. Mean coverage and standard errors are represented by vertical bar plots and error bars, respectively.

For three late‐season sprays in 2024, canopy side as a factor was included in the analysis. ANOVA failed to show significant differences in spray coverage for the canopy side as the main and interaction effects. Numerically lower coverage was quantified in the mid‐section of the bottom canopy zone in PSD‐SSCDS compared with GC treatment during all three spray events. Overall, the least coverage was obtained in the mid‐canopy side of the bottom zone (10.81%), followed by the top zone (13.08%) in PSD‐SSCDS on 25 July. Coverage in the pertinent canopy sections for the same spray event in the GC treatment was 43.62% and 32.68%, respectively (Fig. 9).

### 3.3 Canopy volume

**Figure 10:**
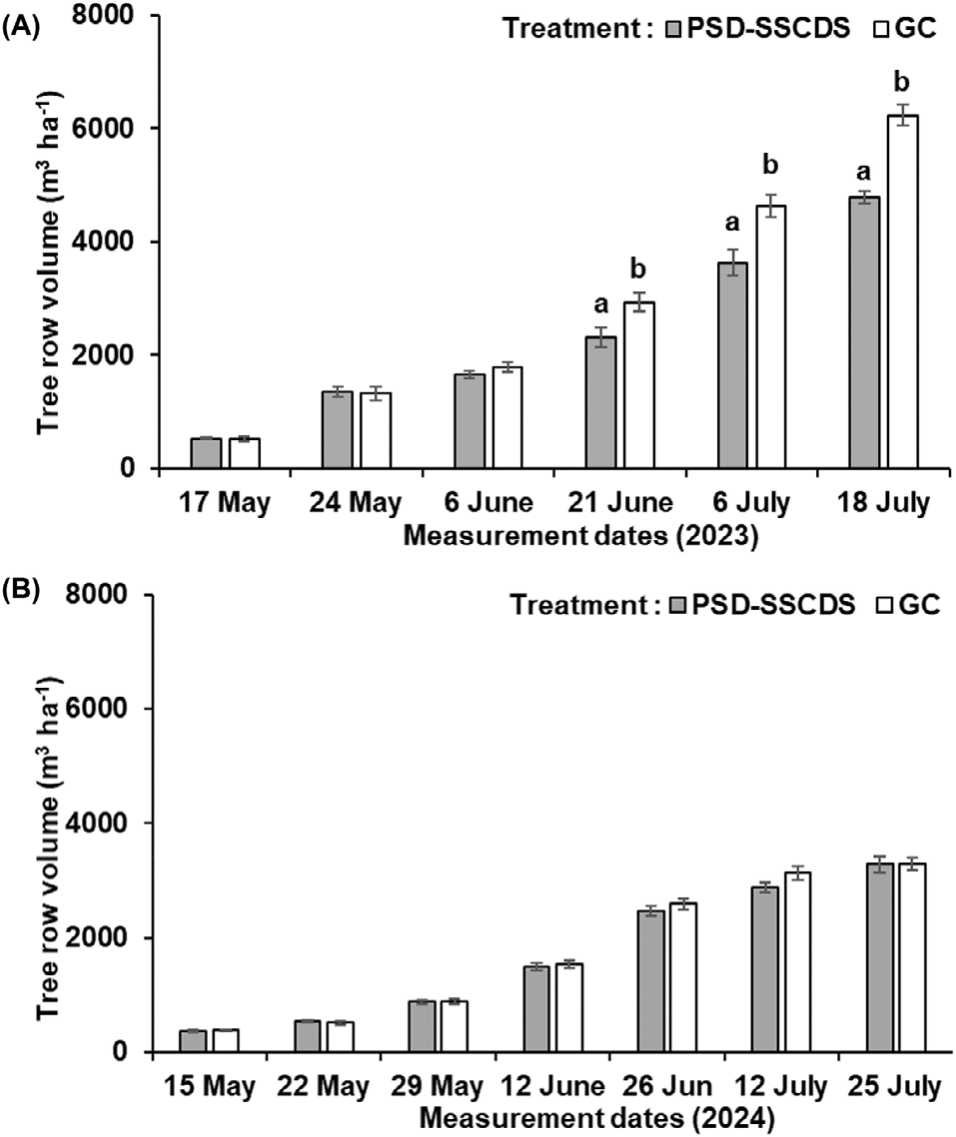
Tree row volume measured during each spray application in pneumatic solid set canopy delivery system (PSD‐SSCDS), and grower control (GC) treatment plots in the 2023 (A) and 2024 (B) seasons. Distinct letters indicate statistically significant differences among treatment means on a particular date at *α* = 0.05, as represented by the two‐sample *t*‐test. The error bar indicates standard error.

There were no significant differences in the overall TRV for PSD‐SSCDS and GC treatments for the initial three spray events (*P* > 0.05) in 2023. However, significant differences between two treatments were observed for the last three spray events conducted after 21 June (*P* < 0.05). These differences were the result of tucking the canopy within trellis wire in the PSD‐SSCDS block before 21 June. There were no significant differences in TRV between the two treatments throughout the 2024 season (*P* > 0.05). Average TRV in the 2023 season for PSD‐SSCDS and GC treatment ranged from 523.08 to 4787.42 m^3^ ha^−1^ and 519.71 to 6234.31 m^3^ ha^−1^, respectively (Fig. 10(A)). The TRV range for the 2024 season was 367.56–3279.28 m^3^ ha^−1^ for PSD‐SSCDS and 380.67–3286.61 m^3^ ha^−1^ for GC (Fig. 10(B)).

### 3.4 Cluster and foliar powdery mildew disease control

**Figure 11:**
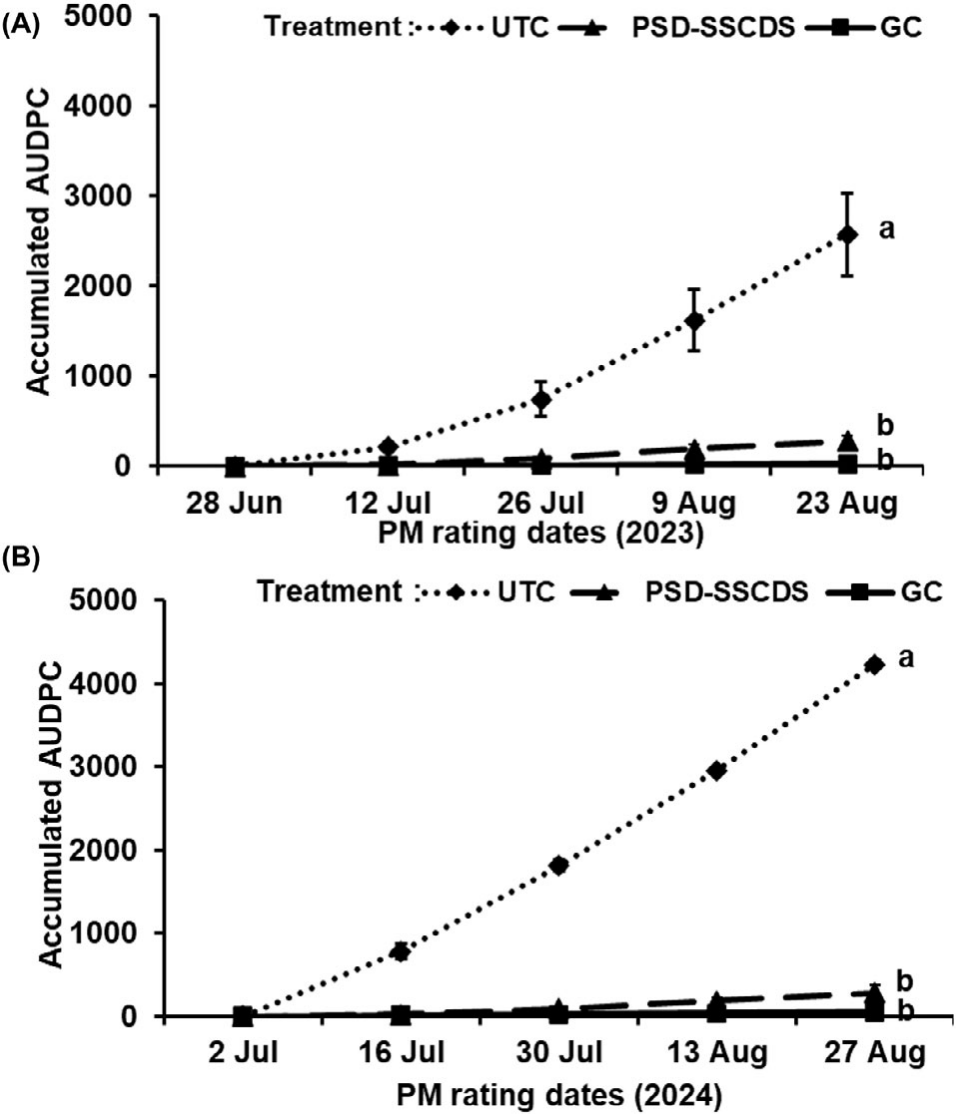
Area under disease progress curve for *Erysiphe necator* severity on clusters among different treatments; untreated control (UTC), pneumatic solid set canopy delivery system (PSD‐SSCDS), and grower control (GC) in the 2023 (A) and 2024 (B) season. Distinct letters indicate statistically significant differences among treatment means at *P* = 0.05, as represented by Tukey's honest significant difference test. The error bar indicates standard error.

As expected with any fungicide treatment, accumulated cluster AUDPC was significantly lower in the PSD‐SSCDS and GC treatments than in the UTC (*P* < 0.0001) in 2023. Overall, cluster AUDPC in the PSD‐SSCDS (262.17) and GC (18.48) treatments was reduced by 89% and 99% (Fig. 11(A)) compared with UTC (2525.01). There was no significant difference in accumulated AUDPC between the GC and PSD‐SSCDS treatments (*P* = 0.7900). These trends were also seen in 2024. Accumulated AUDPC reductions of 93% and 99% compared with the UTC (4233.57) were observed in the PSD‐SSCDS (280.54) and GC (54.58) treatments, respectively (Fig. 11(B)). Statistical analysis confirmed that overall cluster AUDPC in GC and PSD‐SSCDS were statistically comparable (*P* = 0.0800). However, AUDPC in both treatments varied significantly compared with the UTC (*P* < 0.0001). Actual disease severity ratings for clusters are seen in Table 5.

**Table 5:** Powdery mildew disease severity ratings (%, mean ± standard error) on clusters, presented as percent surface area infected at five assessment points per season in untreated control (UTC), pneumatic solid set canopy delivery system (PSD‐SSCDS), and grower control (GC) treatments

Season	Rating dates	UTC	PSD‐SSCDS	GC
2023	28 June	7.4 ± 2.0	0.4 ± 0.1	0.0 ± 0.0
	12 July	22.4 ± 6.0	1.6 ± 0.2	0.1 ± 0.1
	26 July	53.6 ± 14.2	8.0 ± 3.5	0.4 ± 0.1
	9 August	71.4 ± 6.4	7.3 ± 2.6	1.1 ± 0.6
	23 August	65.1 ± 11.9	5.3 ± 2.4	0.3 ± 0.2
2024	2 July	37.9 ± 7.2	0.9 ± 0.4	0.2 ± 0.1
	16 July	73.5 ± 5.0	2.4 ± 0.4	1.3 ± 0.3
	30 July	73.6 ± 7.9	6.8 ± 2.5	0.7 ± 0.1
	13 August	90.1 ± 2.4	6.5 ± 2.6	1.0 ± 0.3
	27 August	92.6 ± 1.8	7.67 ± 4.0	1.6 ± 0.3

**Figure 12:**
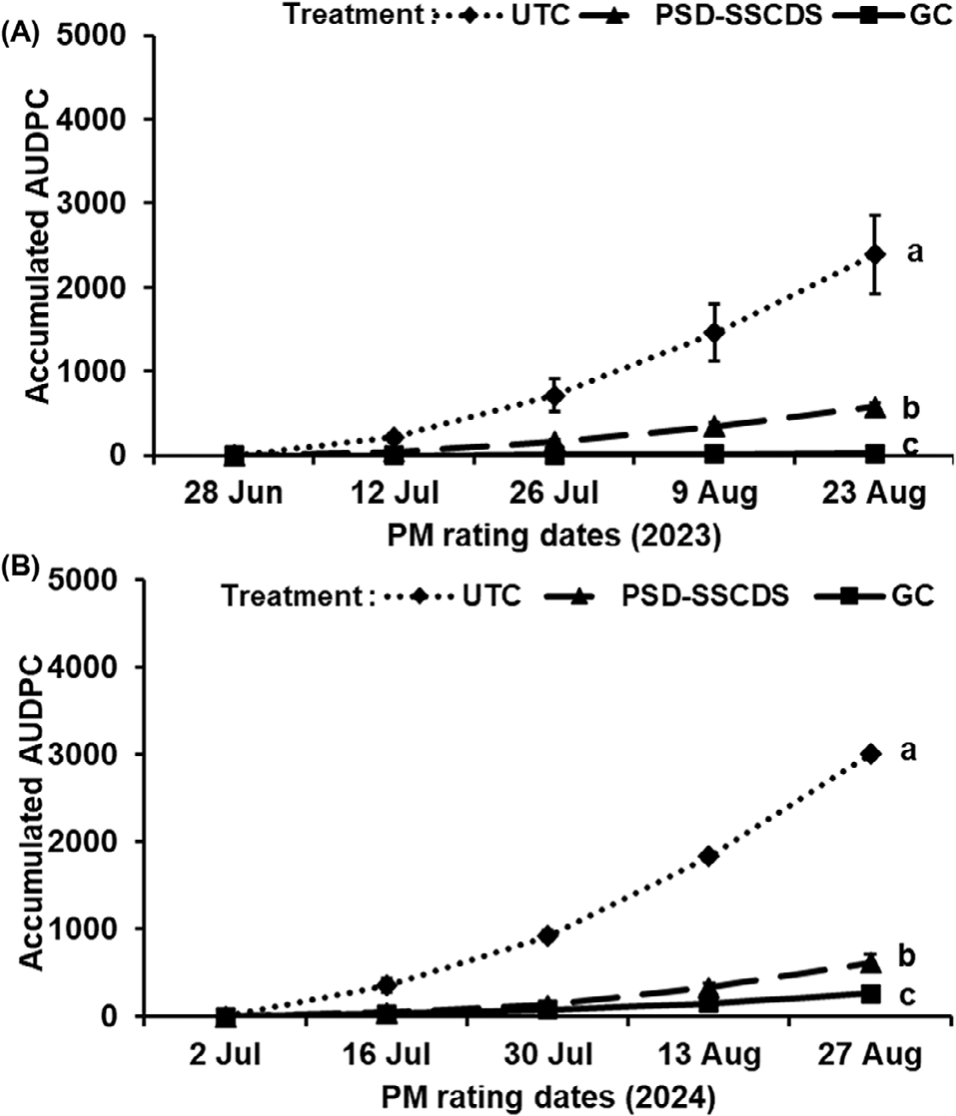
Area under disease progress curve for *Erysiphe necator* severity on leaves among different treatments; untreated control (UTC), pneumatic solid set canopy delivery system (PSD‐SSCDS), and grower control (GC) in the 2023 (A) and 2024 (B) season. Distinct letters indicate statistically significant differences among treatment means at *P* = 0.05, as represented by Tukey's honest significant difference test. The error bar indicates standard error.

Similar to cluster disease severity, the same trends were observed in foliar disease severity. Fungicide applications using the PSD‐SSCDS and GC treatments significantly (*P* < 0.0001) reduced accumulated AUDPC by 76% (592.87) and 99% (18.01), respectively, relative to the UTC (2488.71) in 2023 (Fig. 12(A)). However, statistical analysis revealed significantly higher foliar disease control in GC than in PSD‐SSCDS (*P* = 0.0032). In 2024, this same pattern was observed—both fungicide spray treatments performed better than the UTC (3004.52); however, GC (258.59) had overall better foliar disease control than PSD‐SSCDS (611.90) (*P* = 0.0274) (Fig. 12(B)). GC consistently outperformed PSD‐SSCDS in foliar PM control, resulting in an overall 17.5% lower AUDPC across the two study seasons. Actual foliar disease severity ratings are summarized in Table 6.

**Table 6:** Powdery mildew disease severity ratings (%, mean ± standard error) on leaves, presented as percent surface area infected at five assessment points per season in untreated control (UTC), pneumatic solid set canopy delivery system (PSD‐SSCDS), and grower control (GC) treatments

Season	Rating dates	UTC	PSD‐SSCDS	GC
2023	28 June	8.5 ± 3.2	0.8 ± 0.2	0
	12 July	22.5 ± 2.6	5.7 ± 1.7	0.1 ± 0.0
	26 July	48.1 ± 2.6	10.7 ± 2.4	0.3 ± 0.1
	9 August	59.1 ± 6.2	15.5 ± 2.6	1.0 ± 0.2
	23 August	73.4 ± 2.6	17.8 ± 4.0	0.5 ± 0.1
2024	2 July	18.9 ± 2.0	1.1 ± 0.2	0.3 ± 0.1
	16 July	32.0 ± 1.3	4.5 ± 0.6	4.0 ± 0.7
	30 July	48.2 ± 4.9	8.3 ± 1.7	2.8 ± 0.1
	13 August	82.1 ± 2.3	20.6 ± 4.6	7.3 ± 1.3
	27 August	85.7 ± 2.6	19.7 ± 2.1	8.5 ± 0.9

### 3.5 Effect on cluster and berry weight

**Figure 13:**
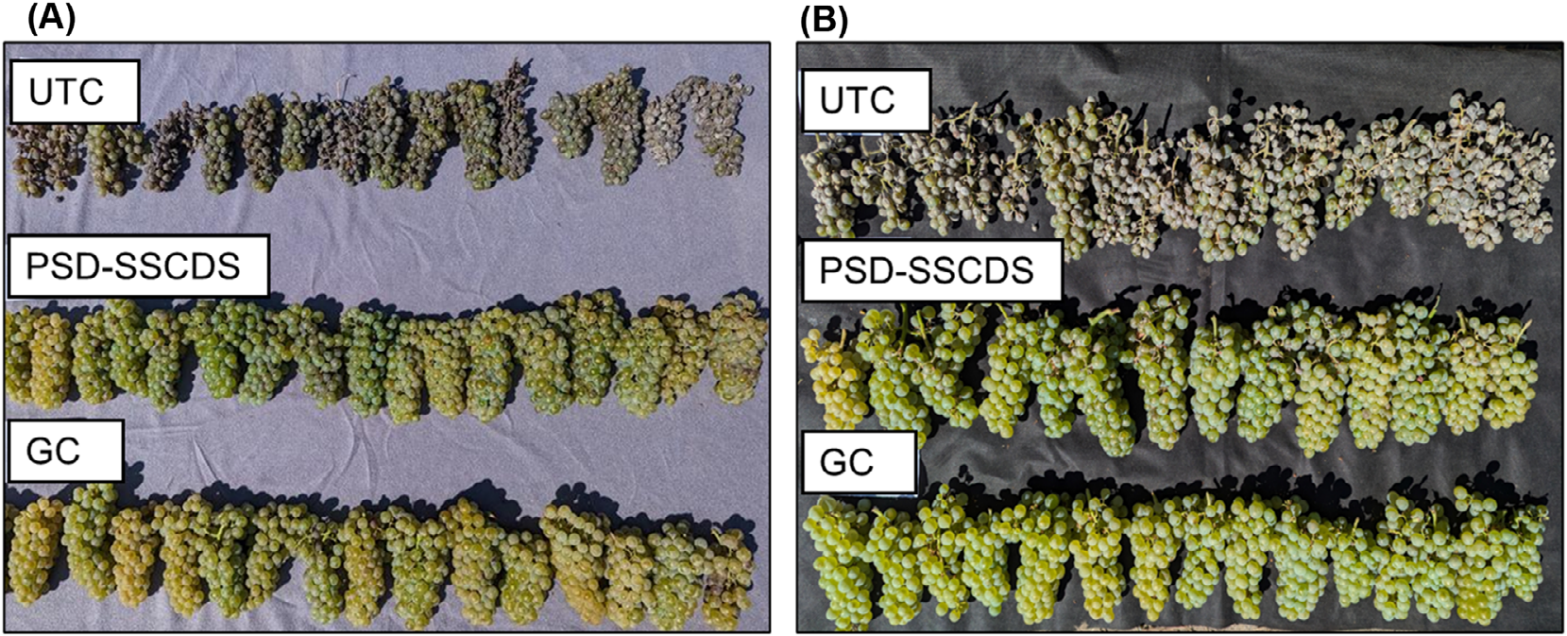
Representation of treatment effects on grape clusters collected randomly from untreated control (UTC), pneumatic solid set canopy delivery system (PSD‐SSCDS), and grower control (GC) treatment plots in the 2023 (A) and 2024 (B) season.

In both growing seasons, significant differences in average cluster weight were observed among treatments (*P* < 0.0001) (Table 7). This was entirely driven by disease status (Fig. 13); the UTC treatment had a significantly lower (*P* < 0.0001) cluster weight than PSD‐SSCDS and GC, with the sprayed treatments being comparable during both study years [*P* = 0.0560 (2023); *P* = 0.7200 (2024)]. Berry weights were also significantly lower in the UTC (*P* < 0.0001) in both growing seasons compared with PSD‐SSCDS and GC (*P* = 0.9990 and *P* = 0.9900).

**Table 7:** Cluster and berry weight (mean ± standard error) measured at harvest in untreated control (UTC), pneumatic solid set canopy delivery system (PSD‐SSCDS), and grower control (GC) treatments

Year/season	Yield parameters	UTC	PSD‐SSCDS	GC
2023	Cluster weight (g)	66.93 ± 4.90 ^a^	150.75 ± 5.38 ^b^	140.62 ± 5.56 ^b^
	Berry weight (g)	0.77 ± 0.04 ^a^	1.33 ± 0.04 ^b^	1.34 ± 0.04 ^b^
2024	Cluster weight (g)	41.41 ± 4.17 ^a^	104.97 ± 6.81 ^b^	106.14 ± 7.29 ^b^
	Berry weight (g)	0.80 ± 0.05 ^a^	1.21 ± 0.04 ^b^	1.29 ± 0.04 ^b^

Distinct letters indicate statistically significant differences among treatment means at *P* = 0.05, as represented by Tukey's honest significant difference test.

## 4 DISCUSSION

Significant differences in spray coverage were observed between the PSD‐SSCDS and airblast sprayer (GC) treatments. Across all spray events in the 2023 and 2024 seasons, significantly higher coverage was achieved in GC than in PSD‐SSCDS. Similar findings were reported by Mozzanini *et al*.^28^ when comparing coverage in SSCDS and airblast sprayers. Despite the higher canopy volume in the GC treatment during late‐season sprays in 2023 (Fig. 10(A)), consistently higher coverage in GC compared with the PSD‐SSCDS treatment was obtained (Fig. 7(A)), which can be attributed to the greater airflow generated by the axial fan, leading to improved spray penetration within the canopy.^29^ This can also be supported by the improved coverage in GC compared with PSD‐SSCDS in mid‐canopy sections of the bottom zone during late‐season sprays in 2024 (Fig. 9). Although the PSD‐SSCDS spray resulted in reduced coverage compared with GC, all treatments except late‐season sprays (7 July 2023 and 25 July 2024) carried out using PSD‐SSCDS achieved overall spray coverage above 15%, which is the minimum threshold required for crop protection.^30^, ^31^, ^32^

Despite low coverage, statistically comparable control of *E. necator* on clusters was observed in the PSD‐SSCDS and GC treatments. This can be explained by the fact that PSD‐SSCDS provided adequate coverage (>15%) during the critical window when berries are most susceptible to *E. necator*, typically from pre‐bloom until about two to four weeks after fruit set.^33^ As the berries age and begin to ripen, they rapidly develop ontogenic resistance, making them increasingly resistant to infection. Inadequate spray coverage was quantified in the fruiting zone of PSD‐SSCDS for the sprays in July, when berries had aged out of this critical window. These findings align with previous research on the biological efficacy evaluation of various SSCDS variants in modern apple orchards and vineyards. Sahni *et al*.^34^ reported lower spray coverage and comparable pest control using PSD‐SSCDS compared with an airblast sprayer in a commercial apple orchard. Two additional bioassay‐based studies reported comparable pest and disease control despite significantly lower coverage in SSCDS than in airblast sprayers.^20^, ^35^ Fungicide applications using PSD‐SSCDS resulted in substantially reduced (78% combined for both seasons) foliar disease progression compared with UTC. However, in both seasons, foliar disease control in the GC treatment was consistently higher than in PSD‐SSCDS. This could be attributed to the overall improved coverage (Fig. 7) and more uniform within‐canopy coverage (Figs 8 and 9) obtained in fungicide applications with the GC treatment. Higher foliar disease severity in PSD‐SSCDS can also be explained by inadequate coverage in the mid‐section of the bottom canopy zone during late‐season sprays. Reduced coverage could have implications for disease management, particularly on basal leaves, canes, and trunk‐adjacent tissues that can serve as inoculum reservoirs for subsequent seasons. However, no increased disease severity was observed on clusters during the second season (2024) study trials. Unlike berries, the canopy as an entity does not develop ontogenic resistance—only individual leaves do—and grapevine canopies have indeterminant growth, producing susceptible tissue all season long. Overall, better disease control was achieved in PSD‐SSCDS for the 2024 season compared with the previous year, which could be due to light canopy hedging of excessively long shoots that was done in 2024. This canopy management allowed the spray to better target the canopy by maintaining the TRV below the maximum canopy size threshold (2573.9 m^3^ ha^−1^)^22^ for PSD‐SSCDS spray applications, potentially reducing the unsprayed canopy volume in 2024 compared with 2023.

Previous studies have reported lower canopy penetration in SSCDS compared with airblast sprayers, particularly on abaxial leaf surfaces.^14^, ^35^ Similarly, Bhalekar *et al*.^22^ reported significantly lower spray deposition on abaxial leaf surfaces compared with adaxial leaf surfaces for the PSD‐SSCDS adopted in the current study. This could also explain lower the disease control on leaves in the PSD‐SSCDS compared with the GC treatment. Beyond powdery mildew, the performance of PSD‐SSCDS against other grapevine pathogens may depend on pathogen‐specific infection sites and spray coverage requirements. Diseases such as downy mildew, which may primarily infect the abaxial leaf surface, could require increased spray penetration and higher underside leaf coverage. Nonuniform deposition across leaf surfaces could still offer better disease control for systemic pesticides, which are absorbed through plant tissues and transported throughout the plant's vascular system. A mechanical solution to improve disease control in vigorous vineyards could be adopting alternative emitters with suitable orientation, spray swaths, droplet size, and uniform spray deposition potential. Post optimization, future research should evaluate PSD‐SSCDS performance against diseases with diverse infection sites and include multi‐disease efficacy trials in commercial vineyards to better define its role as an alternative spray system.

Spatial variability in disease severity is also critical when spray deposition varies within the canopy. In this study, powdery mildew severity was assessed using randomized cluster/leaf sampling across the canopy zones rather than stratified by specific zones (top *versus* bottom or exterior *versus* interior). This approach was adopted because of the relatively smaller and more uniform foliage (effective canopy height 0.9–1.3 m) of the modified VSP‐trained grapevines and to maintain consistency with disease rating protocols used in commercial vineyard assessments.

To make the PSD‐SSCDS system commercially viable, additional information on its adaptability to other training systems or potential uses beyond pest and disease control (e.g., evaporative cooling, frost mitigation) is needed. In addition, SSCDS has proven to be a drift‐reducing and chemical‐saving technology^18^; research is warranted to explore the optimization of PSD‐SSCDS with reduced chemical mixture application rates. With escalating regulatory restrictions on synthetic chemical use and the growing need for sustainable agricultural practices, PSD‐SSCDS, with potentially reduced chemical input, may provide a viable pest management alternative.

## 5 CONCLUSIONS

Compared with the airblast sprayer, spray coverage in PSD‐SSCDS was significantly lower, but still provided adequate control of grapevine powdery mildew on clusters. Additional improvements in disease control for foliar powdery mildew using the existing PSD‐SSCDS variant could be achieved by reducing the unsprayed canopy volume through effective canopy management practices and/or by adopting alternative spray emitters. Biological efficacy results suggest that PSD‐SSCDS can potentially be used for powdery mildew disease management in Washington vineyards, but the adoption economics needs to be considered relative to existing spray application methods.

## CONFLICT OF INTEREST

The authors declare that they have no conflict of interest.

## Data Availability

The data that support the findings of this study are available on request from the corresponding author. The data are not publicly available due to privacy or ethical restrictions.
